# The cost of human papillomavirus vaccination delivery at the administrative and health facility levels in the Philippines

**DOI:** 10.1016/j.jvacx.2024.100459

**Published:** 2024-02-18

**Authors:** Josephine G. Aldaba, Cecilia L. Llave, Ma. Esterlita V. Uy, Kim Patrick Tejano, Ma. Romina C. Aquino, Migel Antonio P. Catalig, Alvin Duke R. Sy, Haidee A. Valverde, Jessica Mooney, Rose Slavkovsky

**Affiliations:** a Institute of Child Health and Human Development, University of the Philippines Manila-National Institutes of Health, Manila, Philippines; b Disease Prevention and Control Bureau, Department of Health, Manila, Philippines; c Department of Physical Sciences and Mathematics, College of Arts and Sciences, University of the Philippines Manila, Philippines; d Department of Epidemiology and Biostatistics, College of Public Health, University of the Philippines Manila, Philippines; e Institute of Health Policy and Development Studies, University of the Philippines Manila-National Institutes of Health, Manila, Philippines; f Center for Vaccine Innovation and Access, PATH, Seattle, WA, USA

**Keywords:** Human papillomavirus (HPV), Immunisation, Vaccine delivery, Costing, Health economics, Low- and middle-income countries (LMICs)

## Abstract

**Background:**

The World Health Organization has recommended the inclusion of human papillomavirus (HPV) vaccines in national immunization programs to address the global problem of cervical cancer. In the Philippines, HPV vaccination was introduced in a phased approach in 2015. This study seeks to estimate the cost of delivery of the HPV vaccination program and its operational context in the Philippines.

**Methods:**

This was a retrospective, cross-sectional micro-costing study focused on ongoing HPV vaccination delivery and its operational context across all levels of the health system. Using structured questionnaires and data collection from secondary sources, the weighted mean financial and economic costs and costs per dose at the national, subnational, and health facility levels were estimated.

**Results:**

The weighted mean financial and economic costs per dose of the HPV vaccination program aggregated across all levels of the health system were $US3.72and $29.74, respectively. Activities contributing most significantly to costs were service delivery and vaccine collection or distribution and storage at the health facility and administrative levels, respectively. The opportunity costs for health worker and non-health worker time accounted for 77% of the economic cost per dose.

**Conclusion:**

The total weighted mean financial and economic costs of HPV delivery are within range of those reported in other countries. Costing studies can help identify cost drivers with local operational context to help inform policymakers and program managers in budgeting and planning interventions to improve program implementation.

## 1 Introduction

In the Philippines, cervical cancer is the second most frequent cancer among women, with about 8,000 new cases per year [1]. Human papillomavirus (HPV) vaccines protect against cervical cancer and are recommended by the World Health Organization (WHO) for inclusion in all national immunization programs [2]. HPV vaccines are also essential to the Global Strategy to Accelerate Cervical Cancer Elimination, which has set the target of providing HPV vaccines reaching 90% of all girls aged 9 to 14 years by the year 2030 [3].

In the Philippines, which is a lower-middle-income country not eligible for support from Gavi, the Vaccine Alliance, the health system is divided into the national level, represented by the Department of Health (DOH); the subnational level, which consists of the 17 regional and 81 provincial offices; and the health facility level. Under the regional health offices are several provincial health offices and under these health offices are several rural or city health offices, which implement the health programs at the municipality and city level, respectively. The national and subnational levels have administrative roles in the system, and the health facilities are the implementers of health programs.

HPV vaccination was first piloted by the DOH in 2013 through a school-based demonstration program targeting girls aged 10 to 14 years in select schools in two regions [4]. In 2015, the National Immunization Program (NIP) began a phased scale-up of HPV vaccination in select provinces in 11 of 17 regions targeting girls aged 9 to 14 years through a health facility–based approach [5], which was subsequently changed back to a school-based approach. In the same year, the NIP, together with the Department of Education, started a school-based immunization program which provided measles-rubella (MR vaccine) and tetanus-diphtheria (Td) vaccines to Grade 1 and Grade 7 students [6]. According to the DOH, since 2020—due to school closures prompted by the SARS-CoV-2 pandemic—HPV vaccination has shifted again to a health facility–based approach and is being provided in 48 provinces and 57 cities, which represent 59% and 39% of the total number of provinces and cities in the country, respectively. HPV vaccination is implemented through a two-dose approach given at least 6 months apart. Although HPV vaccination is being scaled up nationwide, program costs continue to be a concern.

To the authors’ knowledge, there are no published or unpublished studies on the cost of HPV vaccination delivery in the Philippines. Cost estimates from a demonstration project in Vietnam found the economic cost per dose to be $1.92 and $2.08 (2009 US$) for facility- and school-based delivery, respectively [7]. Given the target population for HPV vaccines (girls 9 to 14 years of age) and alternative delivery strategies needed to reach this population, delivery costs of HPV vaccines have been noted to be significantly higher than the delivery costs of routine infant NIP vaccines [8].

This study seeks to estimate the ongoing costs of HPV vaccine delivery in the Philippines and explore the operational context and linkages between delivery costs and program implementation.

## 2 Materials and methods

### 2.1 Study design

We conducted a retrospective micro-costing study to estimate ongoing financial and economic delivery costs of HPV vaccines in the Philippines and also included operational research components to understand the HPV vaccination program’s contextual factors. Ongoing delivery costs are defined as the recurrent costs incurred year-on-year to ensure the availability and continuation of HPV vaccination services and the annualized cost of capital equipment [9]. Financial costs pertain to accounting transactions (i.e., monetary outlays or expenditures), whereas economic costs assess resource-based opportunity costs, regardless of whether a financial transaction occurred [8]. The study methodology was aligned with immunization costing [9] and operational research guidelines [10] and a prior economic and operational context study in six countries [11]. We used school year 2018–2019 as the reference year for the study. This choice reflects the DOH preference for a school-based HPV vaccination strategy and the last time HPV dose 1 and dose 2 were given under the school-based approach.

### 2.2 Sample selection

Sample selection was conducted in two steps. First, we used a purposive but criteria-driven approach to identify provinces or cities representative of the socioeconomic characteristics of the country (i.e., non-rural vs. rural^1^) and also program maturity (i.e., provinces or cities that had introduced HPV vaccination in 2017 vs. those with HPV programs since 2015 or 2016). Only provinces where the HPV vaccination program had been implemented for at least a year prior were considered for inclusion in the study. As a result, no urban localities were included because those classified as strictly urban areas had implemented the program outside of the reference period. In the sampling frame, the four levels (rural, non-rural, early implementer, and late implementer) altogether consisted of 227 municipalities or cities within 15 provinces from 7 regions in the Philippines.

Second, we used EPIC Sample Design Optimizer to randomly select municipalities or cities within the subset of identified provinces [12]. A total of 42 sites (municipalities or cities) were initially selected; these are referred to as health facilities and are the primary unit of analysis. One province from one region and its four municipalities was replaced because initial communication with the provincial health office revealed the province only implemented HPV vaccination in 2019, which was outside the reference period. A total of three other rural health units (servicing three separate municipalities) in two provinces were also replaced because these areas did not implement HPV vaccination during the reference period. Randomized resampling was done where the sample frame allowed. We were not able to collect data from one health facility and one provincial health office because the interviewees were unavailable. The final study sample consisted of 41 health facilities, 15 subnational health offices (7 regional and 8 provincial), and 1 national office.

### 2.3 Data collection

Primary data collection took place July through August 2022 via face-to-face interviews with immunization program staff using a structured questionnaire programmed in Open Data Kit (ODK) and administered with electronic tablets. ODK is an open-source application for data collection using Android mobile devices and data submission to an online server. At the national level, two of the five interviews were done via videoconference. The structured questionnaire focused on HPV vaccination program activities and associated costs, which are listed in Table 1, during school year 2018–2019. Activities evaluated aimed to capture the full scope of the HPV vaccination program. Estimating demand refers to enumerating the target population and crisis management is responding to rumors and investigating any reported adverse events following immunization (AEFIs).

**Table 1:** Human papillomavirus (HPV) vaccine program activities and cost categories evaluated.^1^

	**Cost categories evaluated**									
**HPV vaccination program activities**	**Financial costs** (Direct financial outlays or expenditures for HPV vaccination program)						**Opportunity costs** (Opportunity costs for use of existing resources by HPV vaccination program)			
	Per diems	Meeting costs	Vehicle rental/public transport	Fuel costs (vehicle and other equipment), energy for cold chain equipment, and vehicle maintenance	Radio messages/printing, distribution of IEC materials	Other costs (printing, record copies)	Value of donated goods (donated dry supplies and airtime)	Health worker time	Non-health worker time (DepEd^2^ staff and community stakeholders)	Annualized cost for vehicles and equipment (refrigerators and vaccine equipment)
Vaccine procurement^3^							X	X		
Estimating demand								X		
Program planning and management	X	X						X	X	
Social mobilization and IEC^4^	X	X			X		X	X	X	
Training	X	X						X	X	
Vaccine collection or distribution and storage	X		X	X				X		X
Service delivery^5^	X		X	X				X	X	X
Supervision^6^	X		X					X	X	X
Record keeping						X		X		
Waste management	X		X	X				X	X	X
Crisis management	X					X		X	X	

1 Boxes without an “x” denotes that cost category was not relevant for the corresponding activity.

2 DepEd – Department of Education.

3 National level procurement costs, including the cost of vaccine product, related supplies, and shipping, taxes, and customs clearance are excluded from the main analysis. These costs are included when calculating the cost per dose inclusive of national procurement costs, as stated in the methods.

4 IEC – information, education, and communication materials.

5 Service delivery program activity only for health facility interviews.

6 Data on per diems and transport costs collected only for supervision at administrative levels.

We also extracted data from paper-based or electronic records at health facilities, which captured the number of HPV vaccination sessions conducted, the location and date of each session, and the number of dose 1 and dose 2 vaccinations administered (total and disaggregated by age).

We collected primary and secondary data on unit prices—such as staff salary scales, cold chain equipment, and vehicle and energy costs—from government documents and literature. In addition, we collected information on the number of HPV vaccine doses (and other vaccines included in the NIP) from the DOH and the Field Health Service Information System. Data collection methods are summarized in the Supplemental Appendix.

### 2.4 Data analysis

Operational and costing data were analyzed using Microsoft Excel (Microsoft Corporation, Redmond, Washington, USA) and Stata 14 (StataCorp LLC, College Station, TX, USA) and tabulated for each health facility or program office according to previously published methodological guidelines [9]. All cost data were collected in Philippine peso (PHP) and converted to 2018 US dollars using an exchange rate of 52.66 PHP per USD [13], the average exchange rate in 2018. Data were disaggregated by level of the health system (i.e., facility, regional, and national) and by program activity. For each facility or program office, we estimated financial and opportunity costs by multiplying quantities of each resource used by their unit price to obtain the cost estimates for each HPV vaccination program activity. Where values were unknown, we imputed medians to calculate the cost estimates. For capital items (e.g., vehicles, equipment), we annualized the replacement price over their assumed useful life-year using a 3% discount rate. To allocate shared resources to the vaccination program for costs related to vaccine collection or distribution and storage, we used either a quantity-based proportion or volume-based proportion of HPV vaccines relative to the total vaccines delivered for routine NIP vaccinations. These proportions used to allocate shared resources are detailed in the Supplemental Appendix. For meetings, trainings, and other in-person activities, we used the proportion of the activity that focused on HPV vaccination as reported by respondents during the interviews.

Costs at each level of the health system were disaggregated by type (financial or economic costs), category (e.g., per diems, meeting costs, health worker time), and program activity (e.g., vaccine procurement, training, service delivery). Delivery cost estimates in the main analysis exclude the value of vaccines, supplies, and related shipping, handling, and customs clearance fees. Weighted mean costs were reported per health facility, the volume of which was calculated per the formula below.

$$\mathrm{Volume}\;\mathrm{weighted}\;\mathrm{mean}\;\mathrm{cost}\;\mathrm{per}\;\mathrm{dose}=\frac{\sum_{i=1}^{n}{\mathrm{Cost}}_{i}}{\sum_{i=1}^{n}{\mathrm{Doses}}_{i}}$$

In the formula, *i* represents each site in the study sample and *n* is the sample size for that level of the health system. We calculated and reported a 95% confidence interval. At the subnational program office level, the mean costs per program office (regional and provincial) in the sample were reported; at the national level, the total costs provided by the national program office were reported. We calculated cost per dose at the administrative levels, applying the formula above but using the estimated number of doses delivered within the catchment area of the program office (based on health information system data). For comparison, we also estimated the financial and economic cost per dose aggregated across all levels, inclusive of all national level procurement costs for vaccine products and supplies.

### 2.5 Ethics approval

The study was reviewed and approved by the University of the Philippines Manila-Research Ethics Board (UPMREB 2021-0586-01). Permissions were obtained through the Department of Health and subnational health offices to conduct visits to the study sites.

## 3 Results

### 3.1 Operational findings at the health facility and administrative levels

All 41 health facilities reported giving HPV vaccination at schools during the reference period. There was an average of 22 schools visited by each health facility during the reference year. Only 16 health facilities (39%) co-administered HPV with other vaccines given in schools. In addition, 17 health facilities (41%) reported providing facility-based HPV vaccination, and 5 facilities (12%) also offered HPV vaccination through outreach locations (barangays or communities). Facility- and outreach-based HPV vaccination was provided for students absent or unable to receive the vaccine during school sessions, to administer dose 2, and to reach out-of-school girls.

Data on the number of HPV doses administered during the reference year were unavailable for 6 (15%) of the 41 health facilities. Of the 35 facilities that reported the number of HPV dose 1 given, 13 (37%) were unable to retrieve records for HPV dose 2.

Six of the 35 facilities (17%) that reported the number of doses did not report the number of HPV vaccination sessions during the reference year and only reported aggregated number of doses given. Among the 29 facilities that reported HPV vaccination sessions, there were a total of 1,002 sessions held, for an average of 35 vaccination sessions per health facility.

A total of 20,250 doses of HPV vaccine were administered by the health facilities in our sample, with 1,365 (7%) given at health facilities and 327 (2%) given at outreach sessions. The majority of doses (18,558 or 92%) were given at schools. On average, there were 579 HPV doses administered per health facility.

Other HPV program activities were carried out at the health facility-level during the reference period. The majority of health facilities held planning and management meetings, conducted social mobilization activities, collected vaccines from higher levels of the health system, and held trainings for the HPV vaccination program. These activities are detailed in Table 2.

**Table 2:** Key human papillomavirus (HPV) program activity characteristics at the health facility level (n = 41).

**Program activity**	**Operational findings**
Vaccine collection or distribution and storage	● 35 (85%) health facilities (HF) collected vaccines from higher levels of health system, with a total of 102 trips to collect HPV vaccine and a mean of 3 trips per HF. ● Median round-trip distance was 73 km per trip. ● Most common (94%) mode of transport used by HFs was vehicles owned by the HF.

Program planning and management	● All 41 HFs reported program planning activities, with a total of 241 activities done and a mean of 6 events per HF. ○ Majority were meetings with school representatives (40%) followed by meetings with other groups such as local government officials and parents (31%). ○ 130 (54%) of the events were 1-day meetings, 102 (42%) of the events were <1 day in duration. ○ 153 (63%) events were not focused on HPV vaccination alone. ○ 50 (21%) events were meetings with mostly HF staff.

Social mobilization	● 38 (93%) HFs reported total of 151 sensitization activities and meetings to develop information, education, and communication (IEC) materials, with mean of 4 events per HF. ○ Meetings to develop IEC materials was the most common activity (58%), followed by sensitization meetings (38%). ○ 114 (75%) were half-day events and 78 (52%) were done together with program planning activities. ● 38 HFs (93%) conducted a total 35 additional IEC activities, of which the most common were distribution of IEC materials (31%) and IEC radio messages (29%).

Crisis management	● 31 (76%) of the HFs had plan to address adverse events following immunization (AEFIs). ● Crisis management activities were done by 15 HFs (37%). ○ There were a total 33 response activities with a mean of 2 activities per HF. The most common activity was holding meetings with parents and the community to address rumors related to HPV vaccine causing sterility or infertility and on the side effects of vaccines, which they related to news on Dengvaxia at that time. ● One HF reported a suspected serious AEFI, which necessitated an investigation. It concluded the event was coincidental and not related to the vaccine. ● Health workers and school staff were the main participants in CM activities.

Training	● 28 HFs (68%) had training for HPV vaccination, with a total of 50 trainings and a mean of 2 trainings per HF. ○ Most training sessions (70%) were 1-day events. ○ 32 sessions (64%) were done with trainings on other programs. ● Most common event was training HF staff on HPV vaccination (56%).

Record keeping	● 38 HFs (93%) regularly received and used record-keeping materials for HPV vaccination. ● All HFs collected and maintained data in their HFs and sent them to the next higher level of the health system (provincial health office or regional health office). ● Health worker time was the resource most used.

Waste management	● 33 (80%) of HFs carried out their own waste management activities through burial or use of a septic vault. ● Remaining HFs (20%) sent their sharp-edged waste to the district, provincial, or regional levels for disposal (also through burial).

Supervision	● 37 (90%) HFs received supervision visits, which varied in frequency and number. ● 19 (51%) of these 37 HFs had supervision visits combined with supervision for other immunization programs (infant immunization and school-based immunization). ● 38 (93%) HFs had supervisory or monitoring visits to the schools.

Estimating demand	● All HF were able to mention their target population for HPV vaccination. Most of HFs used a target population from the actual number of enrollees for the reference year.

Vaccine procurement	● 13 (32%) HFs reported purchasing or receiving donations of dry supplies (cotton, alcohol, waste bins) for the reference year. ● Health worker time was the resource most used.

At the subnational level of the system, the mean total number of HPV vaccine doses given was 11,288 doses. Nationally, 170,262 total HPV vaccine doses were given in the Philippines during the reference period. We used these values to compute cost per dose.

Key program characteristics at the administrative levels (subnational and national) are reported in Table 3. The main activities conducted by program offices were vaccine collection or distribution and storage; program planning and management; social mobilization and information, education, and communication (IEC); training; and supervision. Two provincial offices conducted crisis management activities. Only one provincial office conducted waste management (through burial). Vaccine procurement was only done at the national level.

**Table 3:** Key human papillomavirus (HPV) vaccine program characteristics at the subnational and national administrative levels.

**Program activity**	**Regional and provincial levels** **(n = 15 offices)**	**National level (n = 1)**
Crisis management (CM)	● 11 subnational offices (73%) had CM plans in place to manage AEFIs. They follow guidelines from the next higher level of the system. ● Immunization program staff were involved in the planning process for CM plans. ● 2 provincial offices implemented 3 CM response activities. ● All response activities involved holding meetings to discuss misconceptions about HPV vaccination and involved health office staff.	● The national level sets the CM plans and cascades guidelines to lower levels of the system. ● No CM activities specific to the HPV vaccination program took place at the national level during the reference period.

Vaccine collection or distribution and storage	● 13 subnational offices (87%) had HPV vaccines delivered to them from the next higher level of the system. ● 4 subnational offices collected vaccines from higher-level facilities (2 of these also had the vaccines delivered to them). ● Trips to collect HPV vaccines were combined with collecting other vaccines.	● Distribution of vaccines was done by a third-party logistics company that delivered the vaccines to the lower levels of the health system (regional health offices and city health offices). ● Trips to distribute HPV vaccines were sometimes done together with other National Immunization Program (NIP) vaccines.

Waste management	● Only one subnational office did waste management disposal (through burial).	● n/a

Program planning and management	● Done in 12 subnational offices (80%). ● Most common activity at regional and provincial levels was conducting meetings. ● Activities were done between 1 and 12 times per year and involved mostly program staff.	● Planning meetings were done at the national level as well as meetings with lower levels of the system. Each type of meeting occurred 2 times for the reference year.

Social mobilization and information, education, and communication (IEC)	● 9 subnational offices (60%) reported social mobilization meetings and 13 (87%) reported other IEC activities such as printing and distributing IEC materials, media publications, and radio messaging. ● Activities done between 1 and 3 times during the reference year and involved program staff.	● Social mobilization activities at national level included 3 meetings to develop content and IEC materials. ● Other IEC activities included printing and distributing materials and social media engagement. ● There were donations of printed IEC materials.

Training	● Trainings were done in 10 subnational offices (67%). ● Most common type of training was refresher or routine training for current NIP staff. These were combined with trainings for other NIP vaccinations. ● Most trainings were conducted once and primarily involved program staff.	● No training meetings were held during the reference period.

Supervision	● 8 subnational offices (53%) received supervisory visits. ● Frequency ranged from monthly to once per year. ● Supervisory visits were conducted by 13 of the subnational offices (87%).	● Monthly supervisory visits to lower levels of the health system were conducted.

Other activities and human resources	● Other activities included vaccine procurement, estimating demand, and record keeping. ● These subnational offices only requested vaccines but did not actually procure them. ● Human resources were accounted for separately and not as part of each activity.	● Other activities included estimating demand, vaccine procurement, and record keeping. ● HPV vaccines were self-procured. A total of 5 shipments with a total of 500,000 HPV vaccines doses were delivered during reference year. ● Human resources were accounted for separately and not as part of each activity.

### 3.2 Costing findings at the health facility and administrative levels

We estimated the total weighted mean financial and economic costs at the health facility level to be $805 and $11,350, respectively (Table 4). The opportunity cost for health worker and non-health worker time contributed to 87% of the mean economic costs. Fuel, energy, and maintenance costs represented 3% of mean economic costs and the greatest share of financial costs (Fig. 1a). Service delivery was the main activity contributing to both financial and economic costs, with weighted mean financial and economic costs of $473 (59%) and $5,499 (48%), respectively (Supplemental Appendix).

**Table 4:** Weighted mean financial and economic cost of human papillomavirus (HPV) vaccine delivery by health system level in 2018 US dollars.

	Mean financial costs [95% confidence interval]	Mean economic costs [95% confidence interval]
Health facility (n = 41)	$805 [$487–$1,122]	$11,350 [$7,656–$15,043]
Subnational (n = 15)	$20,440 [$5,127–$35,752]	$43,850 [$21,815–$65,884]
National (n = 1)	$21,592	$114,978

**Fig. 1a:**
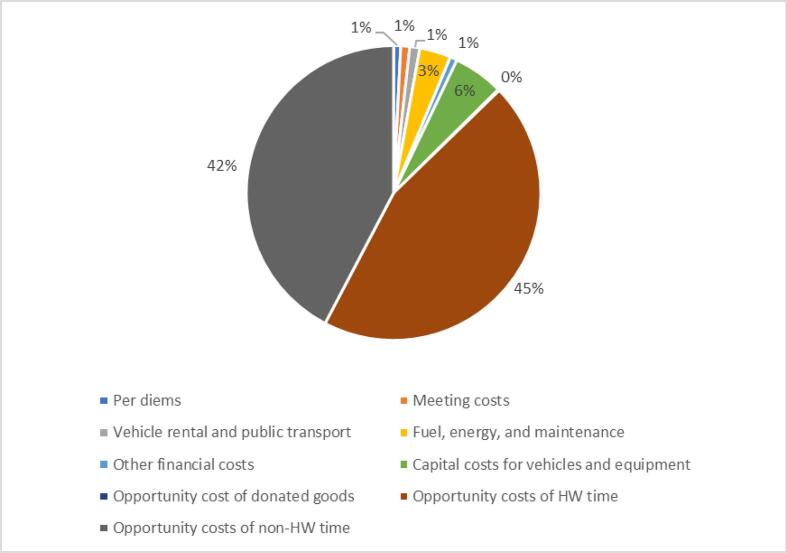
Weighted mean economic costs of human papillomavirus (HPV) vaccination at the health facility level, by cost category (n = 41).

At the subnational level, the total weighted mean financial and economic costs were $20,440 and $43,850, respectively (Table 4). The cost category of capital costs for vehicles and equipment contributed to the largest share (30%) of economic costs. Fuel, energy, and maintenance costs were 25% of economic costs and 53% of financial costs (Fig. 1b). Vaccine collection or distribution and storage was the activity that comprised the greatest share of costs at this level (Supplemental Appendix).

**Fig. 1b:**
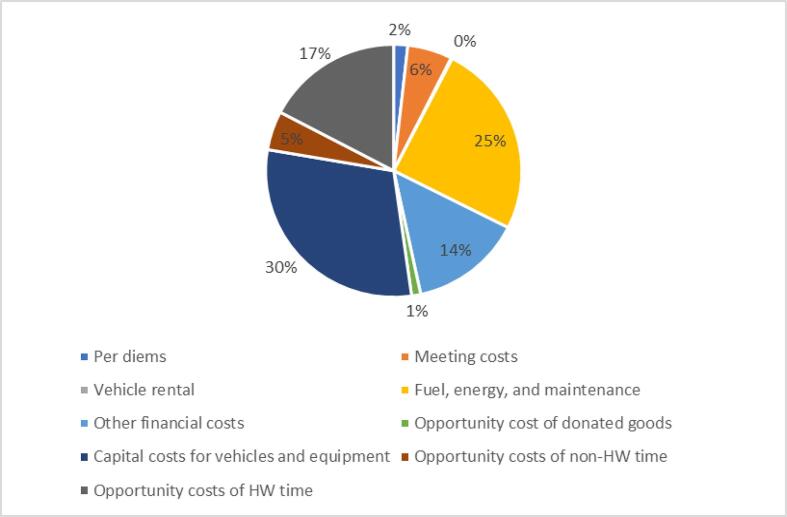
Weighted mean economic costs of human papillomavirus (HPV) vaccination at the subnational level, by cost category (n = 15).

National-level costs were estimated at $21,592and $114,978 for financial and economic costs, respectively (Table 4). At the national level, the largest share of financial costs was for vehicle rental for vaccine collection or distribution and storage at 70%. Capital costs for vehicles and equipment for vaccine collection or distribution and storage contributed the largest share (74%) of economic costs. National-level cost estimates by program activity are further reported in the Supplemental Appendix.

**Fig. 2:**
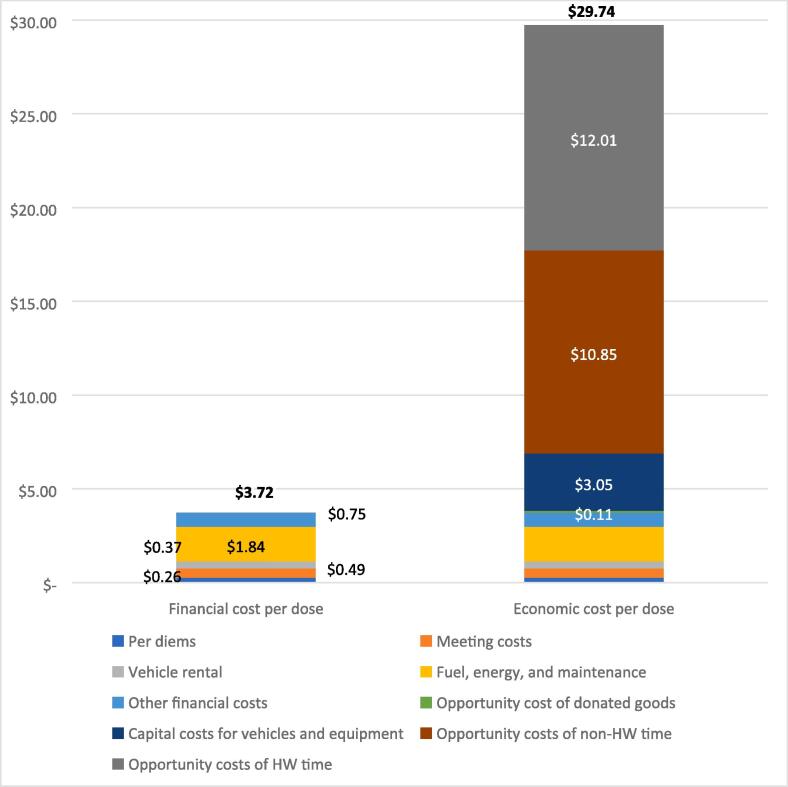
Estimated financial and economic cost per human papillomavirus (HPV) vaccine dose aggregated across all levels of the health system, by cost category in 2018 US dollars.

The financial and economic costs per dose at aggregated across all levels of the health system are reported in Table 5. Across all levels, the estimated financial cost per dose is $3.72 and the economic cost per dose is $29.74 (Fig. 2). Health facility–level costs contribute to 85% of the economic cost per dose, and regional-level costs contribute to 49% of the financial cost per dose. As shown in Fig. 2, fuel, energy, and maintenance costs amounted to 49% of the financial cost per dose aggregated across all levels of the health system, whereas opportunity costs for health worker and non-health worker time accounted for 77% of the total economic costs per dose aggregated across all levels. When national level procurement costs are included, the financial and economic costs per dose increased to $42.20 and $68.22, respectively.

**Table 5:** Estimated cost per dose across all health system levels in 2018 US dollars.

	Financial costs per dose [95% confidence interval]	% of total	Economic costs per dose [95% confidence interval]	% of total
Health facility	$1.78 [$0.64–$2.93]	48%	$25.18 [$8.69–$41.68]	85%
Subnational	$1.81 [$0.27–$3.35]	49%	$3.88 [$1.22–$6.55]	13%
National	$0.13	3%	$0.68	2%
Total aggregated cost per dose across all health system levels	$3.72		$29.74

## 4 Discussion

In school year 2018–2019, the Philippines implemented a school-based delivery strategy for HPV vaccine with delivery costs estimated at $3.72 and $29.74 per dose for financial and economic costs, respectively.

The estimated financial cost per dose in this study is similar to those cited in previous costing studies in other LMICs, while the economic cost per dose is relatively higher but still within range of reported results [7], [8], [11], [14], [15], [16], [17], [18], [19]. The relatively high economic cost per dose may be due to the level of engagement of health staff and non-health staff in service delivery and program planning and management at the health facilities, which accounted for a substantial proportion of the economic cost per dose. When we include national level procurement costs, the cost per dose estimates increases substantially due to the high number—500,000 doses—of vaccines and supplies procured during the reference period relative to the denominator of 170,262 doses delivered in the Philippines.

While our estimates are generally in line with results from other costing studies, they reflect a program context of low coverage. In data obtained from the DOH, national HPV vaccine coverage for the reference year was estimated at 48% for dose 1 and 5% for dose 2. These low rates could have been affected by the domestic controversy in late 2017 over the anti-dengue vaccine Dengvaxia, which was shown to diminish confidence in vaccines from 93% in 2015 (prior to the controversy) to 32% in 2018 [20]. Low HPV vaccine coverage affects the cost of delivery. One recent study in Tanzania found evidence that the drop-out rate from first to second dose and the efficiency of service delivery had the largest impact on costs and concluded that the more girls vaccinated per session, the lower the cost per fully vaccinated girl [19]. We anticipate that increased coverage could lead to a lower cost per dose in the Philippines.

Another opportunity for improving cost efficiency could be switching to a one-dose schedule. In our study sample, among those health facilities that were able to report the numbers of dose 1 and dose 2 given, only 54% of those who received the first dose were able to complete both doses. The WHO Strategic Advisory Group of Experts on Immunization (SAGE) recently recommended a one- or two-dose HPV vaccine schedule for the primary target of girls aged 9 to 14 years [21]. Switching to a one-dose schedule could contribute to savings in resources, improve efficiencies in implementation, and increase HPV vaccine coverage.

When we consider what activities and cost categories are contributing substantially to costs, at the health facility level, the program activity contributing to the greatest share of costs was service delivery. Other substantial cost contributors include vaccine distribution and storage, program planning and management, and social mobilization. These findings are consistent with other costing studies [14], [15], [16], [17], [18], [19]. Opportunity costs of health care worker time were not as prominent at the administrative levels as at the health facility level. This is expected since these health system levels are not involved in direct implementation of health program activities but are more concerned with administrative and support tasks, including serving as vaccine stores for the next level of the health system.

As previously mentioned, most HPV vaccines were delivered in school-based locations. Studies have shown that school-based strategies in HPV delivery achieve greater coverage rates but at greater overall costs compared to facility-based or outreach strategies [8], [22]. In the Philippines, we were not able to compare costs of school-based versus facility-based delivery due to sample size limitations, further we are not able to assess the relationship between cost and coverage in our study. We did observe that only 39% of health facilities carried out HPV vaccine delivery together with other vaccinations, namely, MR and Td vaccines. While we did not ask why a facility did not give the HPV vaccine with the other vaccines, there were open-ended comments that pointed to logistical problems, e.g., HPV vaccines were not yet available during the time of implementation of the other school-based immunizations. Systematically exploring why health facilities do or do not co-administer HPV vaccines with other school-based vaccines is an area for additional research.

When HPV vaccine is delivered alongside other vaccines, only the first dose of HPV vaccine is co-administered. As health worker and non-health worker time represent the majority of service delivery economic costs, the lack of integration—particularly for the delivery of dose 2—diminishes opportunities for economies of scope and may have contributed to higher opportunity costs. One study recommends further evaluation of the workload and cost impacts of combining other health interventions with HPV vaccine delivery, as few studies have examined this [23].

Local costing studies can help budget immunization programs and evaluate the efficiency and cost effectiveness of program strategies [24]. Unfortunately, very few countries have available estimates of the costs of providing immunization services [24]. The Immunization Costing Action Network’s Immunization Delivery Cost Catalogue (IDCC) identifies just 61 unique publications reporting immunization unit costs representing only 33 countries [22]. Moreover, to have relevant and utilizable results in a costing study, data should be complete, available, and as accurate as possible. As far as we know, this is the first immunization costing study in the country. While the local immunization program will benefit from the results, there is room for improvements in reporting of data to support future immunization costing studies.

Our study has limitations. Data collection was focused to the 2018–2019 school year, which was the last time the school-based strategy was used to provide both HPV vaccine doses. The following year, the second HPV vaccination was provided via the health facilities, partly because of lockdowns due to the SARS-CoV-2 pandemic. Hence, data collection was done four years after the referenced HPV activities were conducted, subjecting the findings to possible recall bias.

Moreover, many of the health staff interviewed were new to the program and were not part of the program during the reference year. Nevertheless, given that HPV vaccination program activities are repeated year after year, this routine frequency may help reduce the impact of recall bias on the data.

About a third of the total number of sampled health facilities could not provide the total numbers of HPV vaccine dose 2 given for the reference year. This underestimation would result in a lower reported total number of HPV doses given, which would subsequently lead to a higher estimated cost per dose. Moreover, school-based immunizations are not required to report through Field Health Service Information System, the national health information system that reports data on the health services given from all health facilities in the country. This incomplete reporting was also aggravated by instances of immunization staff turnover, lack of proper endorsements of records from resigning staff, and the lack of an adequate digital record retrieval system.

While 39% of the health facilities in our sample co-administered HPV with other vaccines during school-based vaccination sessions, we were not able to allocate service delivery costs across the different health interventions due to data limitations. Thus, estimates for service delivery costs in this subset of health facilities is likely overestimated for the HPV vaccine program.

## 5 Conclusion

The study estimates the ongoing financial and economic costs per dose for HPV vaccine delivery in the Philippines at $3.72 and $29.74, respectively. Those estimates reflect the activities carried out during the 2018–2019 school year when the HPV vaccine program implemented a predominantly school-based strategy targeting girls aged 9 to 14 years and achieved relatively low coverage.

The cost categories contributing most significantly to total costs at each level of the health system were opportunity costs for health worker and non-health worker time at the health facility level; capital costs for vehicles and equipment at the subnational level; and capital costs for vehicles and equipment at the national level. We anticipate that increasing HPV vaccine coverage and changing to a one-dose schedule could improve the cost efficiency of the program. Our study offers country-level data with localized context to help inform policymakers and program managers in budgeting and planning interventions to improve program implementation in the Philippines.

## Conflict of interest

CLL has accepted speaking and research engagements for both GSK and MSD. The rest of the authors declare that they have no known competing financial interests or personal relationships that could have appeared to influence the work reported in this paper.

## Funding

This work was funded through PATH by a grant from BMBF (German Federal Ministry of Education and Research) administered through KfW development bank. This work was also supported, in whole or in part, by the Bill & Melinda Gates Foundation [INV-005630]. Under the grant conditions of the Foundation, a Creative Commons Attribution 4.0 Generic License has already been assigned to the Author Accepted Manuscript version that might arise from this submission.

## CRediT authorship contribution statement

**Josephine G. Aldaba:** Conceptualization, Data curation, Formal analysis, Investigation, Methodology, Project administration, Supervision, Validation, Writing – original draft, Writing – review & editing. **Cecilia L. Llave:** Conceptualization, Formal analysis, Investigation, Methodology, Project administration, Writing – original draft. **Ma. Esterlita V. Uy:** Conceptualization, Investigation, Methodology, Project administration, Supervision. **Kim Patrick Tejano:** Conceptualization, Investigation, Methodology, Project administration, Resources, Supervision. **Ma. Romina C. Aquino:** Data curation, Investigation, Methodology, Project administration, Supervision, Validation. **Migel Antonio P. Catalig:** Data curation, Formal analysis, Software, Validation, Writing – original draft. **Alvin Duke R. Sy:** Data curation, Formal analysis, Software, Validation, Writing – original draft. **Haidee A. Valverde:** Data curation, Investigation, Methodology, Project administration, Supervision, Validation. **Jessica Mooney:** Conceptualization, Formal analysis, Funding acquisition, Methodology, Project administration, Resources, Supervision, Writing – original draft. **Rose Slavkovsky:** Conceptualization, Data curation, Formal analysis, Funding acquisition, Methodology, Resources, Visualization, Writing – original draft, Writing – review & editing.

## Declaration of Competing Interest

The authors declare the following financial interests/personal relationships which may be considered as potential competing interests: Cecilia L. Llave reports a relationship with GSK and MSD that includes: consulting or advisory and speaking and lecture fees.

## Data Availability

Data will be made available on request.

## Footnotes

1 In the Philippines, the designation of urban or rural is done at the level of the barangay (or community). A group of barangays make up either a municipality or a city. A geopolitical area is classified as municipality or city depending on its population and annual income. Both municipalities and cities can have a mixture of rural and urban barangays. In the Philippines, as of 2020, only the cities in Metro Manila and two other cities outside of this have 100% of their barangays classified as urban. These cities were not part of the sample due to the timing of HPV vaccine implementation.
