# Supplementary material for: The cost of human papillomavirus vaccination delivery at the administrative and health facility levels in the Philippines
Source: Vaccine X. 2024 Feb 18;17:100459. doi: 10.1016/j.jvacx.2024.100459 (PMC10900773; doi:10.1016/j.jvacx.2024.100459)
Supplement: Supplementary data 1 [file mmc1.docx]

Supplementary Appendix

Supplementary Table 1. Data collection methods used by health system level.

|  | Health facility level  (n=41) | Subnational level  (n=15) | National level  (n=1) |
| --- | --- | --- | --- |
| Primary data collection via interviews |  |  |  |
| Structured questionnaires covering costing and operations components of the study | x | x | x |
| Secondary data collection |  |  |  |
| Data extraction on vaccination sessions from paper-based records | x |  |  |
| Extraction of vaccination data from national electronic databases and from the Department of Health for the subnational and national levels (when available) |  | x | x |
| Literature (including grey literature) and internet search for unit prices to use in the cost analysis: salaries, per diem rates/allowances, purchase price of capital equipment, etc. | x | x | x |

Supplementary Table 2. Unit costs used in the analysis (in 2018 US dollars)

| **Item category** | **Unit cost used in analysis (in US dollars)** | **References** | **Remarks** |
| --- | --- | --- | --- |
| **Energy prices** |  |  |  |
| Electricity price per kilowatt hour | $0.186 | [1–8] | Value for electricity cost per kilowatt hour was the median of available electricity rates from seven provinces included in the study sample in December 2022. The rates were adjusted for inflation to 2018 PHP then converted into 2018 US dollars. |
| **Equipment and vehicles** |  |  |  |
| Ambulance | $28,485 | [9–26] | Values used was the median of published costs per vehicle type and model. The costs were referenced from local websites of the vehicle manufacturers and car dealerships. |
| 4-wheel-drive truck | $19,180 |  |  |
| Car | $18,439 |  |  |
| Motorcycle | $1,158 |  |  |
| Van | $15,439 |  |  |
| Refrigerators (range) | ($280 to $5,000) | [27] | Values assigned based on make and model reported in questionnaire. |
| **Allocation factors used for human papillomavirus (HPV) vaccine versus other vaccines** |  |  |  |
| Quantity-based proportion (mean and range) | 0.027 (0.011 to 0.085) | Primary data extracted from health facilities and health offices in study sample and [28] | The quantity-based proportion calculates the number of HPV vaccine doses delivered among the total doses delivered for routine NIP vaccines during the reference period. Quantity-based proportions were used to allocate human resources time for vaccine collection and distribution activities. |
| Volume-based proportion (mean and range) | 0.157 (0.064 to 0.529) | [28–29] | The volume-based proportion calculates the volume (cm3) of HPV vaccines delivered among the volume of all routine NIP vaccines during the reference period. Volume-based proportions were used to allocate the annualized capital and energy costs for equipment such as cold chain equipment. |
| **Monthly salaries** |  |  |  |
| *Health workers* |  |  |  |
| 1. Nurse (range)  Salary Grade 11-19 | ($400.78 to $842.76) | [30] | Value used was the average of the salary grades for a particular person type. Salary grades were reported during interviews (primary data collection). |
| 2. National Immunization Program Coordinator (range)  Salary Grade 16-19 | ($629.25 to $842.76) |  |  |
| 3. Physician/Municipal Health Officer (range)  Salary Grade 22-26 | ($1,175.31 to $1,843.66) |  |  |
| 4. Midwife (range)  Salary Grade 8-15 | ($319.41 to $574.37) |  |  |
| 5. Sanitary Inspector  Salary Grade 8 | $319.41 |  |  |
| 6. Cold Chain Manager  Salary Grade 16 | $629.25 |  |  |
| 7. Pharmacist  Salary Grade 15 | $574.67 |  |  |
| 8. Driver  Salary Grade 3 | $232.90 |  |  |
| 9. Administrative Staff  Salary Grade 6 | $280.32 |  |  |
| *School staff* |  |  |  |
| 1. Teachers (range) Salary Grade 11-13 | ($400.78 to $479.45) | [30-31] | Value used was the average of the salary grades for each person type. Salary grades were assumed based on staff type reported in questionnaire. |
| 2. Administrators (range) Salary Grade 19-22 | ($842.67 to $1,175.31) |  |  |
| *Local government unit officials and workers* |  |  |  |
| 1. Local Government Worker  Salary Grade 3 | $232.90 | [30] | Salary grade was assumed based on staff type reported in questionnaire. |
| *Other non-health worker salaries (volunteers)* | $32.97 | Personal communication from HAV from an unpublished study data |  |
| **National level procurement costs** |  |  |  |
| Vaccine product, inclusive of shipping, customs clearance, and taxes | $12.91 per dose | Personal communication from DOH | 500,000 doses received during the 2018-2019 reference period |
| Syringe | $0.05 | Personal communication from DOH | Assumed 1 per vial and an additional 1% fee for insurance for cost calculations |
| Safety box | $14.24 | Personal communication from DOH | Assumed 1 per 100 syringes and an additional 1% fee for insurance for cost calculations |

References for unit costs:

1. Kalinga-Apayao Electric Cooperative, Inc. [cited 01 February 2023]. Available from: <https://www.kaelco.com.ph/>.
2. Central Pangasinan Electric Cooperative, Inc. [cited 01 February 2023]. Available from: <https://cenpelco.com/>.
3. Nueva Ecija II Electric Cooperative, Inc. (Area 1.) [cited 01 February 2023]. Available from: <https://neeco2area1.com/unbundled-power-rates>.
4. Masbate Electric Cooperative, Inc. [cited 01 February 2023]. Available from: <https://www.facebook.com/OfficialMASELCO/>.
5. Antique Electric Cooperative, Inc. [cited 01 February 2023]. Available from: <https://anteco.com.ph/index.php/disclosures>.
6. Iloilo III Electric Cooperative, Inc. [cited 01 February 2023]. Available from: <https://ileco3.com/>.
7. Negros Occidental Electric Cooperative, Inc. [cited 01 February 2023]. Available from: <http://www.noceco.ph/rates.html>.
8. Philippine Statistics Authority. Consumer Price Index and Inflation Rate. [cited 01 February 2023]. Available from: <https://psa.gov.ph/price-indices/cpi-ir>.
9. Honda: The power of dreams. [cited 09 February 2023]. Available from: <https://hondaph.com/>.
10. Kawasaki Motors Philippines. [cited 09 February 2023]. Available from: <https://kawasaki.ph/>.
11. Manila Motor. [cited 09 February 2023]. Available from: <https://manilamotor.com/>.
12. Philcarprice.com. [cited 09 February 2023]. Available from: <https://philcarprice.com/>.
13. Philkotse.com. [cited 09 February 2023]. Available from: <https://philkotse.com/>.
14. Philtoyota.com. [cited 09 February 2023]. Available from: <https://philtoyota.com/>.
15. Toyota Motor Philippines Corporation. [cited 09 February 2023]. Available from: <https://toyota.com.ph/>.
16. Autofun: Find your perfect car. [cited 09 February 2023]. Available from: <https://www.autofun.ph/>.
17. Autoindustriya.com. [cited 09 February 2023]. Available from: <https://www.autoindustriya.com/>.
18. CarGuide.ph: Your guide to the Philippine car industry. [cited 09 February 2023]. Available from: <https://www.carguide.ph/>.
19. Carmudi Philippines. [cited 09 February 2023]. Available from: <https://www.carmudi.com.ph/> .
20. Foton Motor Philippines. [cited 09 February 2023]. Available from: <https://www.foton.com.ph/>.
21. Hyundai Motor Philippines. [cited 09 February 2023]. Available from:  [https://www.hyundai.com/ph/en](%20https://www.hyundai.com/ph/en).
22. Mitsubishi Motors: Drive your ambition. [cited 09 February 2023]. Available from: <https://www.mitsubishi-motors.com.ph/>.
23. MotoDeal. [cited 09 February 2023]. Available from: <https://www.motodeal.com.ph/>.
24. TopGear Philippines. [cited 09 February 2023]. Available from: <https://www.topgear.com.ph/>.
25. Webike Philippines. [cited 09 February 2023]. Available from: <https://www.webike.ph/>.
26. Zig Wheels. [cited 09 February 2023]. Available from: <https://www.zigwheels.ph/>.
27. World Health Organization. PQS Catalogue. Product List, Refrigerators and freezers. [cited 01 March 2023]. Available from: <https://apps.who.int/immunization_standards/vaccine_quality/pqs_catalogue/categorypage.aspx?id_cat=17>
28. Philippine Department of Health. *2018 Annual Report: Field Health Services Information System.* 2018. Available from: <https://doh.gov.ph/sites/default/files/publications/FHSIS_Annual_2018_Final.pdf>.
29. Gavi. *Detailed product profiles (DPPs) for WHO prequalified vaccines.* February 2023. Available from: <https://www.gavi.org/news/document-library/detailed-product-profiles>.
30. Office of the President of the Philippines. *Executive Order No. 201: Modifying the salary schedule for civilian government personnel and authorizing the grant of additional benefits for both civilian and military and uniformed personnel.* 19 February 2016. Official Gazette. Available from: <https://www.officialgazette.gov.ph/2016/02/19/executive-order-no-201-s-2016/>.
31. *DepEd Teachers Salary Grade- SSL Third Tranche (2018).* Government PH. Available from: <https://governmentph.com/deped-teachers-salary-grade-ssl-third-tranche-2018/>.

Supplementary Table 3. Cost of human papillomavirus (HPV) vaccine delivery by program activity at the health facility level, in US dollars.

|  | Financial costs | | Economic costs | |
| --- | --- | --- | --- | --- |
| Program activity | Total weighted financial costs  [95% confidence interval] | % of total^a^ | Total weighted economic costs  [95% confidence interval] | % of  total^a^ |
| Vaccine procurement | $23.88  [$5.84–$41.91] | 3% | $34.39  [$11.78–$56.99] | 0.3% |
| Program planning and management | $17.84  [$0–$36.14] | 2% | $2,581  [$0–$5,384] | 23% |
| Social mobilization and information, education, and communication (IEC) | $94.98  [$0–$218] | 12% | $1,089  [$0–$2,318] | 10% |
| Training | $49.38  [$0–$104] | 6% | $312  [$77.20–$548] | 3% |
| Vaccine collection or distribution and storage | $120  [$55.56–$183.87] | 15% | $640  [$480–$799] | 6% |
| Service delivery | $473  [$199–$747] | 59% | $5,499  [$3,658–$7,339] | 48% |
| Record keeping | $25.18  [$16.26–$34.11] | 3% | $944  [$171–$1,718] | 9% |
| Crisis management and response | $0.85  [$0–$2.02] | 0.1% | $226  [$89.04–$363] | 2% |
| Total | $805  [$487–$1,122] |  | $11,350  [$7,656–$15,043] |  |

* Program activities estimating demand, supervision, and waste management did not have any related costs at the health facility level.

^a^ Does not total to 100% because of rounding off.

Supplementary Table 4. Weighted mean economic costs of human papillomavirus (HPV) vaccine delivery at the subnational and national levels per program activity, in US dollars.

|  | **Subnational level** | | **National level** | |
| --- | --- | --- | --- | --- |
|  | Total weighted economic costs [95% confidence interval] | % of total^a^ | Total economic costs | % of total^a^ |
| Vaccine procurement | N/A |  | $0 | -- |
| Vaccine distribution and storage | $24,251  [$5,998–$42,504] | 55% | $101,956 | 89% |
| Social mobilization | $6,694  [$0–$15,092] | 15% | $6,002 | 5% |
| Program planning | $2,652  [$289–$5,014] | 6% | $4,695 | 4% |
| Training | $1,957  [$0–$4,486] | 4% | $0 | -- |
| Supervision | $455  [$74.67–$835] | 1% | $0 | -- |
| Human resources | $7,596  [$273–$14,919] | 17% | $2,324 | 2% |
| Record keeping | $206  [$0–$506] | 0.5% | $0 | -- |
| Crisis management | $39.74  [$0–$90.59] | 0.1% | $0 | -- |
| Total costs | $43,850  [$21,815–$65,884] |  | $114,978 |  |

^a^ Does not total to 100% because of rounding off.
